# Decline of recent seabirds inferred from a composite 1000-year record of population dynamics

**DOI:** 10.1038/srep35191

**Published:** 2016-10-17

**Authors:** Liqiang Xu, Xiaodong Liu, Libin Wu, Liguang Sun, Jinjun Zhao, Lin Chen

**Affiliations:** 1 School of Resources and Environmental Engineering, Hefei University of Technology, Hefei, Anhui 230009, China; 2 Institute of Polar Environment, School of Earth and Space Sciences, University of Science and Technology of China, Hefei, Anhui 230026, China; 3 Xisha Deep Sea Marine Environment Observation and Research Station, South China Sea Institute of Oceanology, Chinese Academy of Sciences, Sansha, Hainan 573199, China

## Abstract

Based on three ornithogenic sediment profiles and seabird subfossils therein from the Xisha Islands, South China Sea, the relative population size of seabirds over the past 1000 years was reconstructed using reflectance spectrum. Here we present an apparent increase and subsequent decline of seabirds on these islands in the South China Sea. Seabird populations peaked during the Little Ice Age (LIA, 1400–1850 AD), implying that the cool climate during the LIA appears to have been more favorable to seabirds on the Xisha Islands in the South China Sea. Climate change partly explains the recent decrease in seabird populations over the past 150 years, but the significant decline and almost complete disappearance thereof on most of the Xisha Islands is probably attributable to human disturbance. Our study reveals the increasing impact of anthropogenic activities on seabird population in recent times.

Seabirds forage at sea and breed on land, delivering significant quantities of marine-derived nutrients and contaminants to terrestrial ecosystems^1^. Activities of seabirds such as Little Auks and Northern Fulmars in the Arctic resulted in the accumulation of guano, and significantly impacted on the properties of soil and lacustrine sediments, as well as plant communities^2^,^3^,^4^. Similar processes have also been observed in coastal areas and islands worldwide^5^. Moreover, seabirds are quite sensitive to environmental changes and thus are an ideal indicator for variability in climatic systems^6^,^7^,^8^.

The Xisha Archipelago (Fig. 1) is a group of coral islands located in the central South China Sea, and was formed during the mid-late Holocene^9^ (last ~5000 years). It can be divided into two groups: the eastern Xuande Archipelago and the western Yongle Archipelago^10^. Seabirds play a central role in the dynamics of these coral island ecosystems, and studies of their ecology in the South China Sea will contribute to a greater understanding of the development of tropical insular ecosystems^11^. However, the influences of climatic or environmental changes on local seabirds are poorly understood to date. Moreover, as disturbances from human activities have become more frequent in recent years, the anthropogenic influence on fragile coral island ecosystems has drawn increasing attention. A study by Braje and Erlandson demonstrated that humans accelerated extinction of animals and plants^12^. Indeed, human-environment interactions and ecosystem processes in the Anthropocene Era have been listed as priority research topics in palaeoecology^13^.

The Xisha Islands was once a renowned seabird colony, providing habitats for over 60 bird species, and also an important resting place for migratory birds^14^. Major Xisha seabird groups include Sulidae, Terns, Frigatebird and Ardeidae, with Red-footed booby (*Sula sula*) the dominant species^14^,^15^. Specifically, there are >35500 pairs of Red-footed Boobies living on the Xisha Archipelago at present. This is approximately 10% of the world population for this species, rendering it the largest habitat for Red-footed Boobies in the Pacific and second largest in the world^16^. Plants on these islands, including shrubs and trees, in addition to sand grounds, provide habitats for those birds^17^. However, according to our field investigations in 2008 and 2015, birds have almost completely disappeared from these islands with the exception of Dongdao Island. Significant decline in seabirds has also been observed by other investigators. For example, it was found that the present number of seabirds on these islands has declined significantly compared to the year 1926^15^. The population size of some species in the present day may be only 10% of their historical values, and colonies have been greatly reduced in size, or even eliminated^16^,^18^. However, the reason for decline on this scale is still unclear. Though few birds have been observed during field trips, large quantities of avian biological remains, e.g. guano, bones, eggshells, were found in the ornithogenic sediments from the islands. Those materials provide specific insights into seabird ecology over the past 1000 years.

Three sediment cores, ZS2, BD1 and CH, were retrieved from Zhaoshu, Beidao and Chenhang Islands in the Xisha Archipelago, respectively (Figs 1 and 2). In the present study, we attempt to reconstruct seabird population history on these islands during the last millennium, with the primary objective of decoding the possible relationship among climate change, anthropogenic activity and seabird population dynamics.

## Results

### Chronology

Both ^210^Pb and ^14^C dates were utilized to generate a Bayesian age-depth model of each profile using the Bayesian software Bacon^19^. Vertical distributions of excess ^210^Pb in the surface sediments of each profile are provided in Supplementary Fig. S1. A constant initial concentration (CIC) model was used to calculate ^210^Pb dates for profiles BD1 and ZS2, and a constant rate supply (CRS) model was employed for the age calculation of profile CH^20^. Using the dataset created by Reimer *et al.*^21^, the Accelerator Mass Spectrometry (AMS) ^14^C ages of bird bone samples were calibrated into Calendar Years Before Present (Cal BP). During calculation, ∆R (the difference between the regional and global marine ^14^C age) was assumed as −25 ± 20 yr, and the marine carbon component as 100%. Results of radiocarbon dating for the three sediment cores are given in Table 1. All the ^210^Pb ages and AMS ^14^C calibrated dates and the chronology of the cores CH, ZS2 and BD1 are plotted in Fig. 3. According to the age models in Fig. 3, the cores span similar periods, and the sediments we collected were accumulated within approximately the last 1000 years, which includes the whole Little Ice Age (1400–1850 AD).

### Changes in seabird population over the last 1000 years

According to both field investigation and laboratory analyses, the composition of the source materials of the ornithogenic coral sand sediments is very simple. Sediments in deeper parts of the cores are primarily composed of guano and coral sand, with little plant humus, while the surface layers consist mainly of guano, coral sand and plant residue^11^,^22^. The spectral properties of these three end members, i.e. guano, coral sand and plant residue, are quite different (Supplementary Fig. S2), and this enables the use of reflectance spectroscopy to reconstruct their ratios in the sediments. The spectral signal of samples is a mixture of the characteristic spectra of each endmember. Spectral mixing modeling can be employed to identify the contribution of pure guano spectra to the mixed spectra, allowing calculation of the percentage of guano in soils and sediments. Following Liu *et al.*^23^ and Xu *et al.*^24^, we used an interval of 0.01 and synthesized a total of 10 000 spectra curves, covering the guano, plant and coral sand proportions in the range from 0 to 100%. For each spectrum, we chose the percentage value with the lowest standard deviation as the best estimate of the guano proportion for the sample. Linear mixing modeling was employed to estimate the guano proportion in the ornithogenic samples of the CH, ZS2 and BD1 cores, and the relative changes in guano proportion, or relative seabird population, versus time are plotted in Fig. 4a,b.

Importantly, the three islands share an initial increase but subsequent decline in seabird populations over the last 1000 years. These historical changes in relative seabird abundance suggest that these populations may have been subject to the same environmental factor(s).

An assemblage of avian bio-elements in ornithogenic sediments is a reliable marker for seabird population^11^,^25^. However, in the case of surface sediments from the Xisha Islands, the seabird population size based on elemental analysis may be overestimated, due to plant development in recent times. Nutrient-rich guano has a sustainable ability to support vegetation via *in situ* nutrient cycling in the soil. It is likely that the development of vegetation has resulted in remigration of the bio-elements after their deposition, and these elements may be further enriched in humus in the surface sediments^11^,^24^. Nonetheless, our approach works well for sediments receiving little impact from vegetation. Concentration-versus-depth profiles of bio-elements P, Cu, Zn, Cd, As and Se in ZS2 profiles are plotted in Supplementary Fig. S3 and they show a broadly similar down-core distribution. We performed primary component analysis (PCA) on these bio-elements. These elements clustered into one group (Supplementary Fig. S4), and the first principle component (PC1) was identified as a proxy for seabird abundance. Generally, for those sediments where impact from plants was minor, the down-core record of relative seabird abundance from bio-elements was consistent with that obtained from spectrum analysis (Fig. 5). These results further support the premise that the changes in seabird guano proportion calculated from reflectance spectroscopy are a reliable record of historical seabird populations on the three islands.

From the seabird population records reconstructed by reflectance spectrum (Fig. 4a,b), it may be observed that seabird populations on the three islands over the last millennium exhibit some island-specific features. For example, the seabird population record for Beidao (BD1) shows a spike at approximately 1600 AD, while there is no clear equivalent in ZS2 or CH. Though the reason for this remains unclear, migration between islands might be a possible explanation, as Zhaoshu Island is only four kilometers away from Beidao Island. Despite such slight differences, the seabird population records on the three islands exhibit some markedly similar characteristics. In general, the overall occupation history of seabirds on the Xisha Islands over the past 1000 years shows an apparent increase and subsequent decline (Fig. 4a,b). The populations were relatively low during the period from 1000 to 1400 AD, and peaked between 1400 to 1850 AD. The seabird populations indicated by ZS2 and BD1 have been declining since approximately the 1700s, while the CH data suggest an abrupt decrease in the seabird population beginning at around 1850 AD. This difference in onset of decline could be explained by ecological heterogeneity. There are arbors and shrubs on Chenhang Island, but the vegetation on Zhaoshu and Beidao Islands consists only of shrubs. The ecosystem of Chenhang Island is thus more stable and has a higher resilience to disturbance than the other two. Therefore, decline of seabirds on CH lagged behind that of ZS2 and BD1. In addition, historically there have been fewer fishermen on the Yongle Archipelago (where Chenhang island is located), than the Xuande Archipelago (where Zhaoshu and Beidao Islands are located), implying a stronger human impact on ZS2 and BD1. This may also have been responsible for the earlier decrease in seabird populations on Zhaoshu and Beidao Islands than on Chenhang Island.

Seabirds have almost disappeared from most of the islands now. Our recent study also revealed a rapid decrease in seabirds on a different island in the Xisha Archipelago in recent times^26^, implying a common pattern throughout the Xisha Islands. The recent decline in seabird ecology is consistent with our field observations: the number of seabird remains, i.e. bone, eggshell and guano particles, decreases abruptly in the surface samples. A decrease in seabird population was also observed by earlier investigations^15^,^18^,^27^, in good agreement with our reconstructed record.

## Discussion

### Impact of natural forces on seabird population

On the whole, the number of seabirds on the Xisha islands was relatively low before 1300 AD, and exhibits a marked increase between 1300 and 1400 AD. The island ecosystems were initially fragile, and could not support a large number of birds. It is likely that the environment of these islands between 1300 and 1400 AD became stable and suitable for seabird survival. This, in combination with a favorable climate, resulted in an increase in seabird population from 1300 to 1400 AD. The subsequent Little Ice Age (LIA) was a widespread global climate cooling event, and occurred in China and the Northern Hemisphere from approximately 1400 to1850 AD. The Earth surface temperature of both the northern hemisphere^28^ and China^29^ during the LIA was at a lower level than at any time during the past two millennia (Fig. 4c,d). Our research suggests that the seabirds on the Xisha Islands were most abundant during the LIA, with a smaller seabird population during the comparatively warmer period prior to 1400 AD (Fig. 4a,b). Thus, a relatively cool climate appears to be favorable for tropical seabirds on the Xisha Islands. This is in contrast to seabirds in Antarctica, where penguin population sizes were relatively large during warm periods^25^,^30^.

Climate changes could exert an indirect effect on seabird abundance by influencing ice coverage in polar regions, surface marine primary productivity or food availability. For example, climate change impacts retreat/expansion of ice, which then influences penguin population and their distribution^30^,^31^,^32^,^33^. Direct effects of climate change on seabirds may also play a part in population shifts through changes in chick survival^34^. The Xisha Archipelago is located in a tropical area, however, so seabird population changes cannot be explained by changes in ice coverage. It may be inferred that seabird population growth and decline on the Xisha Islands are driven by different factors. A modern survey of the dominant seabird species of the Xisha Archipelago, the Red-footed Booby (*Sula sula*), showed that food availability was the primary factor in increasing breeding success^16^. Marine primary productivity determines the abundance of zooplankton and richness of a variety of prey for seabirds, ultimately influencing seabird population size. Both observed and simulated data have demonstrated that primary productivity in surface waters of the South China Sea is negatively correlated with sea surface temperature (SST)^35^,^36^. From their examination of *Porites* coral records, Wei *et al.*^37^ reported that the monthly summer SST in the Xisha Archipelago approximately 540 years ago (during the LIA) was ~1 °C lower than that at present. Consequently, the relatively low SST during the LIA might have generated a high surface primary productivity and sufficient food for seabirds, which in turn enhanced breeding success and ultimately supported more seabirds.

El Niño-Southern Oscillation (ENSO) is also an important influencing factor on seabird populations. According to Shiozaki and Chen^36^, the SST from 1997–2010 in the South China Sea was positively correlated with the El Niño 3.4 Index, and phytoplankton abundance was affected by the wind mixing intensity, which was determined by the ENSO teleconnection. Furthermore, Song *et al.* reported that high/low El-Niño frequencies corresponded to weakened/strengthened winter monsoons in the South China Sea^38^. For example, the El Ninõ years 1997–98 were marked by a weaker winter monsoon and significantly lower surface primary productivity in the South China Sea^39^. The high SST and weak winter monsoon during an ENSO weakens upwelling currents and reduces seawater mixing, thus interrupting nutrient cycling and reducing surface primary productivity. For example, from the “Medieval Warm Period” (MWP ~ 800–1300 AD) to the LIA, the Siberian High, generally indicative of the East Asian winter monsoon, gradually strengthened^40^, in contrast with the weakening of the summer monsoon calculated from stalagmite δ^18^ O^41^ (Fig. 4e,f). According to the reconstruction of multidecadal-scale rainfall and the Southern Oscillation Index (SOI) in the Xisha Archipelago (Fig. 4g,h), Yan *et al.*^42^,^43^ found the MWP was characterized by less rainfall and more El Niño-dominated conditions, whereas during the LIA, more La Niña-dominated conditions prevailed, corresponding to the peak period of seabird populations (Fig. 4a,b). This result suggests that the low El-Niño frequency during LIA was favorable for seabird breeding on the Xisha Islands.

Rainfall may also affect seabird population, as heavy precipitation can occasionally increase breeding failure of seabirds, for instance penguins^34^. However, the rainfall record reveals that the Xisha Islands received more precipitation, and the number of seabirds was relatively high, during the cool LIA. Unlike in Antarctica, shrubs and trees are abundant on the Xisha Islands. The wet conditions during the LIA would have favored plant development and thus increased habitat availability, facilitating seabird inhabitation.

Changes in solar irradiance of the tropical Pacific over the past 1000 years^44^ could be the ultimate controlling factor. Low irradiance during the LIA (Fig. 4i) induced a low temperature and a stronger East Asian winter monsoon. Those processes, in combination with low El-Niño frequency during the LIA, elevated marine productivity in the South China Sea and prey availability for seabirds. Though Chinese fishermen discovered the Xisha Islands more than 2000 years ago^45^, only a very limited number of people visited there seasonally. Significant impact from humans in the preindustrial period seems unlikely. In summary, seabird populations in this area before 1850 AD were principally controlled by natural factors.

### Impact of human activity on seabird population

The notable decrease in seabird numbers at ~1850 AD (shown as arrows in Fig. 4a,b) to the lowest level identified in our research may be attributed in part to recent global warming. For example, the reconstructed SST data from Xisha *Porites* coral indicates that the late 20th century was warmer (by approximately 1 °C) than the early 20th century^46^. The recent increasing SST in the South China Sea generally corresponded to weaker winter monsoons. Based on coral records, winter monsoon wind velocity appears to have followed a persistently declining trend throughout the entire 20th century^47^,^48^. Moreover, Song *et al.*^38^ reported that 70.2% of El Niño events (from 1854 to 1996) occurred during the weak period of the winter monsoon. In accordance with historical trends, the recent increase in SST, decline in winter wind velocities and increase in El-Niño events should inevitably have caused a decline in seabird populations on the Xisha Islands.

Though climate change can partly explain the decrease in seabird population since 1850 AD, the most important factor impacting on seabird population may have changed. In contrast to natural factors, the stress on ecosystems from human disturbance has been direct and much more severe. Significant anthropogenic activities on the islands began in the mid-19th century, and humans have exerted an increasing impact on such islands since the beginning of the 20th century^45^,^49^. The sharp decline and almost complete disappearance of seabirds on the Xisha Archipelago over the past 150 years was more likely caused by significant and disruptive anthropogenic activity. This finding is supported by modern surveys of seabirds in the Xisha Archipelago by Cao *et al.*^15^, who suggested that the seabird numbers in 2003 and 2004 had declined greatly, as compared to 1926 levels, due to increasing human disturbance. These disruptive anthropogenic activities in the South China Sea island ecosystem may be summarized as follows:

#### Habitat destruction

Large animals introduced into this area, i.e. cows, dogs, cats etc., have destroyed native vegetation on the islands. Human-introduced rodents, i.e. rats, have also been observed on the Xisha Islands by scientists^50^, and this may further account for seabird population decline. At the same time, introduced plants, including *Morinda citrifolia* and *Cocos nucifera* (coconut palm), have caused strong interspecies competition with native plants, subsequently decreasing the availability of the dominant habitat of the Red-footed Boobies (*Sula sula*) on the Xisha Archipelago^16^. Furthermore, large-scale human activities, such as house construction, deforestation, guano mining and farming, have also destroyed substantial seabird habitat. In addition, egg collection by local fishermen in recent times may have proved fatal to bird survival. Habitat destruction may have affected seabirds on all the Xisha islands. However, Zhaoshu and Beidao Islands could have suffered greater human impact in history than Chenhang Island, as fewer fishermen have built houses on Yongle Archipelago than on Xuande Archipelago. This could also partly explain why the decrease in seabird population occurred earlier in ZS2 and BD1 than CH.

Long-term monitoring suggests that coral reefs have suffered a dramatic decline over the past 50 years, due to intense anthropogenic activities^51^. A study of coral reefs in Xisha also revealed a sharp decrease in both living and dead coral coverage^52^. This would have had a significant negative effect on the seabird habitat and on foraging in the South China Sea.

#### Overfishing

As discussed above, lack of marine organism abundance significantly restricts seabird expansion. Myers and Worm reported the rapid worldwide depletion of predatory fish communities^53^. They found that industrialized fisheries typically reduced community biomass by 80% within 15 years of exploitation, and estimated that large predatory fish populations in 2003 were at only approximately 10% of pre-industrial levels. Statistical data reveal that total production from marine fisheries in each of the eight Asian countries around the South China Sea has been increasing since 1950 AD^54^. A study by Christensen *et al.*^55^ also revealed a marked decline in fishery stock throughout the South China Sea, including the Xisha area, since 1960 AD. Though the number of some varieties of tern feeding on small fish may not have been affected by fishery activities, many predatory seabird groups other than terns (Sulidae, frigatebirds, etc.), could have been affected by such human activity. At the same time, overfishing affects reproduction of fish, and may lead to continuous decline of fishery resources and recession of marine ecosystems. In addition, illegal and destructive fishing, such as the use of explosives, bottom trawling and cyanide fishing, would be fatal to fish of all sizes. This could also have led to a decrease in fish resources and the continued decline in seabird populations on these islands.

#### Pollution

Both localized pollution on the islands and more general pollution of the marine environment have occurred. For example, some studies have suggested that soil and ground water in the Xisha Archipelago have been severely contaminated with heavy metals such as Cu, Pb, Hg, Ag and As^56^,^57^. This contamination may have threatened the ecological health of the island ecosystem and may also be responsible for the decline in seabird abundance. In addition, increasing growth of the Asian economy has resulted in the emission of a large number of heavy metals such as Hg and Pb, which has also led to increased pollution of the marine environment via large-scale atmospheric circulation. This part of heavy metals can be ultimately enriched in bird tissues through biomagnification, representing a potential threat to seabirds on the islands. For instance, Xu *et al.* reported that oceanic Hg was significantly enriched in eggshells and that eggshell Hg had increased over the past 700 years, in particular over the past two centuries, in this area^58^. Oil spills are another threat to birds, and Cao *et al.* found that some Red-footed Boobies in the Xisha Archipelago had died from oil pollution^15^. Overfishing and oceanic pollution could affect all the islands almost equally, due to a common foraging area among seabirds and the tendency for islands in the Xisha Archipelago to receive similar levels of heavy metal contamination, for example Hg^57^, and oil pollution^15^.

In summary, unprecedented human activities have significantly affected the independence and integrity of fragile coral island ecosystems. We believe that the sharp deterioration in seabird numbers in the Xisha Islands in the South China Sea over the past 150 years can be attributed primarily to the significant increase in recent anthropogenic activities.

The decline in seabirds on the Xisha Islands is not a unique case. Rawlence *et al.* have also reported dramatic population extinctions and range retraction for birds soon after human arrival on South Island, New Zealand^59^. A review of landbird extinctions in the Pacific Islands suggested a very high extinction rate after human colonization during the Late Holocene period^12^. Briefly, anthropogenic factors have impacted increasingly on seabird population since 1850 AD. Human activity is, and will continue to be, the principal limiting factor for seabird populations on the Xisha Archipelago in the near future. The extinction risk of seabirds on the Xisha Islands is extraordinarily high, and special efforts are now required to protect birds on these tropical coral islets. The loss of seabirds in recent years has been significant, to the extent that the seabirds on the Xisha Archipelago may disappear completely in the near future. We should now devote special efforts to biological conservation on these tropical coral islets in order to protect such birds.

## Methods

### Sample collection

**Figure 1:**
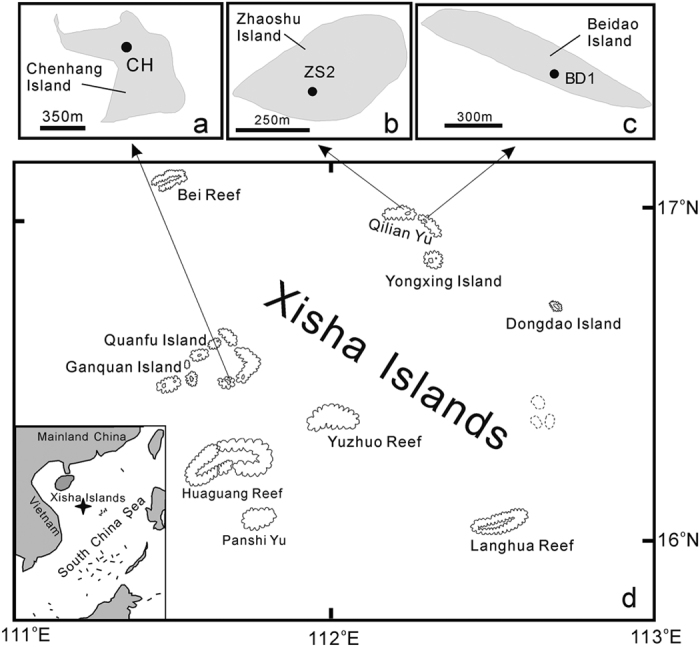
Map of the study area showing sampling sites (modified from ref. 11). Cores CH, ZS2 and BD1 were collected from Chenhang (**a**), Zhaoshu (**b**) and Beidao (**c**) Islands (shaded area in each rectangle), respectively. Star in the map shows location of the Xisha Islands (**d**) in the South China Sea. Filled black circles represent locations of sampling sites. [Reprinted from Chemical Geology, 286(3), Xu, L. Q., Liu, X. D., Sun, L. G., Yan, H., Liu, Y., Luo, Y. H. & Huang, J. Geochemical evidence for the development of coral island ecosystem in the Xisha Archipelago of South China Sea from four ornithogenic sediment profiles, 135–145, Copyright (2011), with permission from Elsevier].

Three well-preserved ornithogenic sediments cores were collected from the Xisha Islands. Profile CH was retrieved from Chenhang Island in the Yongle archipelago in 2008; and cores ZS2 and BD1 were collected from Zhaoshu and Beidao Islands in the Xuande archipelago in 2015. The three profiles were collected from under woodland and shrubs on the islands, at locations given in Fig. 1. When sampling, we inserted 11 cm diameter PVC plastic gravity pipes into the soft substrate and then excavated sediments around the pipes to retrieve the cores. An overview of these three sediment profiles is given in Table 2. A large number of seabird/fish remains, including bones and guano pellets, were observed in the ornithogenic sediments. The CH profile was sectioned at intervals of 1–2 cm *in situ*, while cores ZS2 and BD1 were sectioned in the laboratory at intervals of 1 cm, and subsamples were then homogenized with a pestle and mortar, after being dried at a temperature of 105 °C and passed through a 200-mesh sieve.

**Figure 2:**
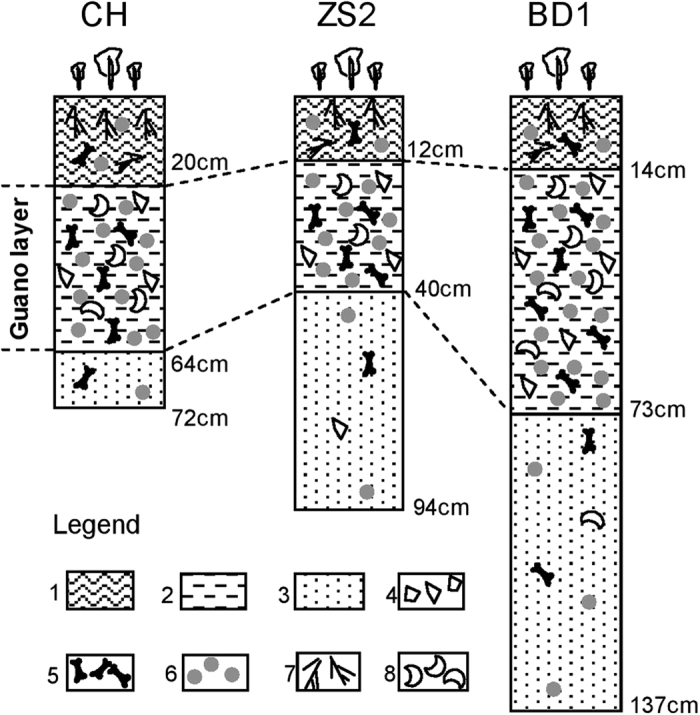
Lithology of the profiles CH, ZS2 and BD1. Legend: 1 grey black humus, plant residues and medium to large size coral sands in the upper layer, a little bit guano and seabird bone remains; 2 light brown ornithogenic sediments, containing a lot of medium to large size coral sands, guano particles, bone remains and fish scales; 3 yellow to white coral sand sediment layer with low organic matter content, a few guano pellets and numerous calcareous bioclasts; 4 calcareous bioclasts; 5 bird and fish bones; 6 guano; 7 remains of plant leaf, stem and root; 8 fish scales.

**Figure 3:**
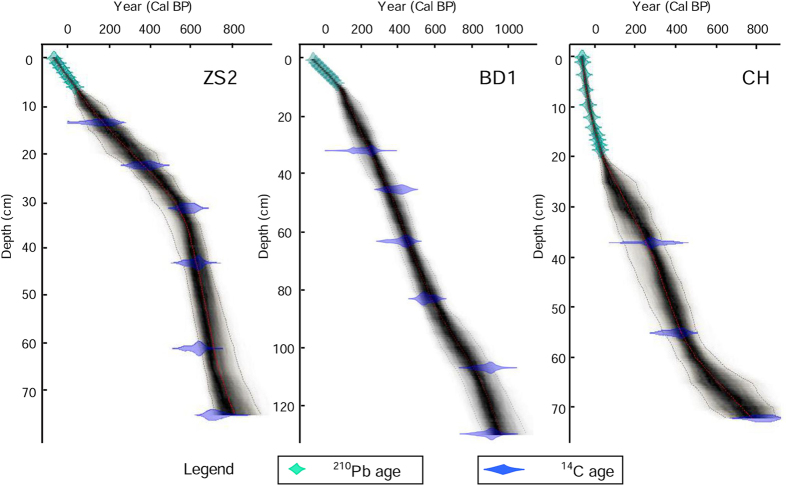
Bayesian age-depth models of the studied profiles ZS2, BD1 and CH by both ^210^Pb and ^14^C dates.

**Figure 4:**
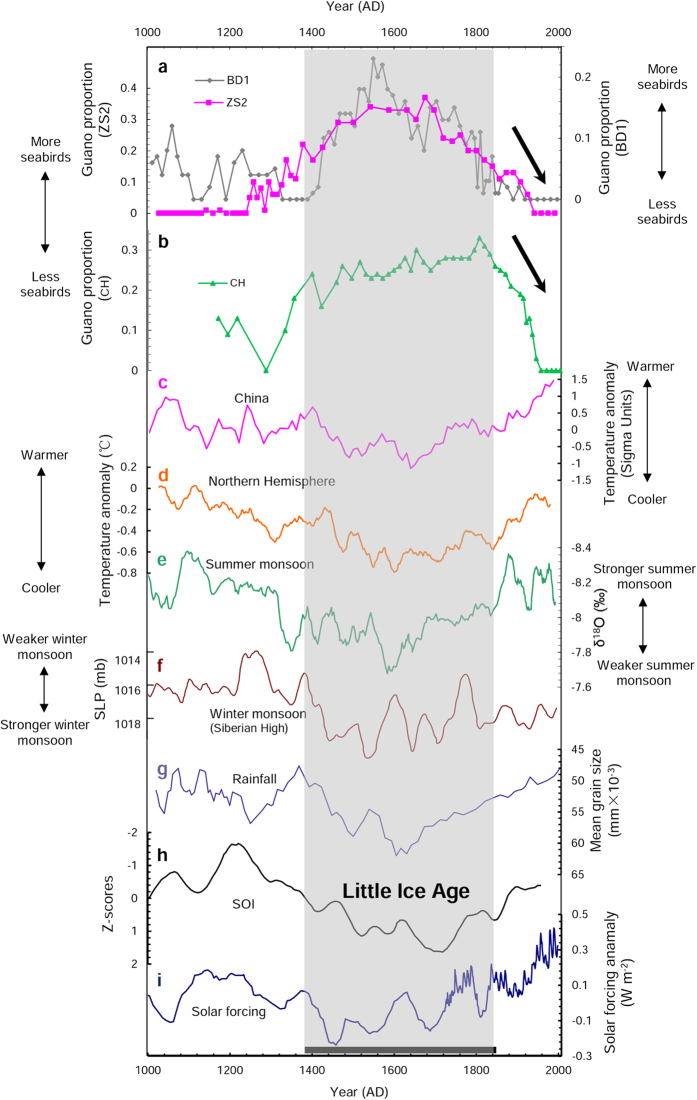
Comparison among long-term seabird population dynamics on the Chenhang, Zhaoshu and Beidao Islands and a variety of climatic records. Cited literature: (**a**) Historical seabird population records on the Zhaoshu and Beidao Islands (this study). (**b**) Historical seabird population record on the Chenhang Island (this study). (**c**) Standardized temperature record in China^29^ showing the relative amplitude of temperature change, but does not provide numerical values of temperature variation. (**d**) Temperature record (30-yr running average) in the northern hemisphere, and temperature anomalies are relative to the 1961–1990 average^28^. (**e**) East Asian summer monsoon record^41^. (**f**) Atmospheric sea-level pressure (SLP) record (Siberia High indicator)^40^. (**g**) Grain size-based historical rainfall record on the Xisha Archipelago^42^. (**h**) A SOI record from Yan *et al.*^43^ (**i**) solar forcing of the tropical pacific over the past 1000 years^44^.

**Figure 5:**
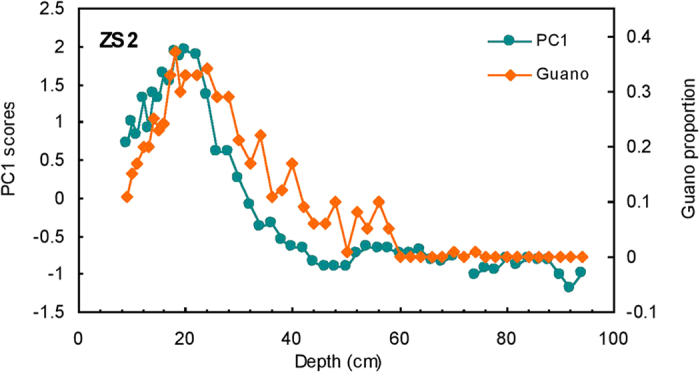
Comparison of reconstructed seabird population records by reflectance (shown as guano) and bio-elements (shown as PC1) for the profile ZS2.

The lithology of the studied profiles is shown in Fig. 2. From this figure, it may be seen that the three cores have an almost identical lithology, and each of the profiles can be divided into three sediment units. There is an evident “guano layer” in each profile, which contains quantities of seabird remains, such as guano particles, eggshells and bones (Fig. 2). Above this guano layer, the sediments are composed of black humus and numerous plant remains, with few bird remains. In contrast, the sediments below the guano layer mainly consist of white coral sand, with only a few scattered guano pellets. Similar lithologies were observed in cores collected from the other four islands of the Xisha Archipelago, i.e. Ganquan, Guangjin, Jinqing and Jinyin Islands, as reported in our earlier study^11^. This similarity in lithology for cores collected from the different islands implies a common development process in the Xisha Archipelago.

### Analytical methods

The ornithogenic coral sand sediment was a mixture of three end members (coral sands, guano and plant residues). To reconstruct concentration-versus-depth profiles of guano, we analyzed the diffuse reflectance spectrum of each bulk sediment. The powdered sample was first packed into a measuring cell, and then the reflectance spectrum of each sample was acquired on a Shimadzu DUV-3700 UV-Vis-NIR recording spectrophotometer, by scanning from 380 to 2500 nm at intervals of 1 nm. Thus, 2 121 data points were obtained for a single spectrum. An external, reference standard polyethylene (zero absorbance) was read alternately with the samples. The resultant reflectance (r) data were then transformed to absorbance (log 1/r) data using UV. Prove software. Spectral analysis was performed at the Public Experimentation Center, University of Science and Technology of China. The spectral signal of ornithogenic sediments is a combination of the characteristic spectrum of each end member. Thus, a linear mixing modeling was performed to estimate the guano proportion in the ZS2, BD1 and CH ornithogenic samples^24^. A higher guano proportion indicated more seabirds occupying the island studied.

For comparison, we also determined levels of several elements, including Cu, Zn, Cd, P, As and Se in the bulk sediments. These elements have been identified as avian “bio-element” indicators for bird droppings in the Xisha Archipelago, and thus may be used to estimate changes in seabird populations^11^. The down-core distributions of the elements P, Cu, Cd, Zn, As and Se were determined for the profile ZS2. The concentrations of As, Se, Cu, Cd and Zn in the sediments were determined by inductively coupled plasma-mass spectrometry (ICP-MS) after digestion with multi-acids at ALS Chemex (Guangzhou) Co. Ltd., while the P level was determined by inductively coupled plasma-atomic emission spectrometry (ICP-AES) at the Institute of Polar Environment, University of Science and Technology of China.

### Dating

The chronology of each profile was established by both ^210^Pb dating and radiocarbon analysis. For ^210^Pb analysis, the upper sediments of all the cores were dated using ^210^Pb activity measured by HPGE gamma spectrometry (manufactured by Ortec, USA), and the lower parts of the ZS2, BD1 and CH cores were dated using AMS ^14^C ages obtained from seabird bones. Specifics of the ^210^Pb test were reported in our earlier study^22^. Radiocarbon analyses of several bird bone samples in each profile were performed at Beta Analytic Inc., or the University of Georgia. The AMS ^14^C dates were then calibrated into Calendar Year Before Present (Cal BP), where the “present” is defined as 1950 AD. In the current study, we established the age models of the three cores using Bayesian analysis.

## Additional Information

**How to cite this article**: Xu, L. *et al.* Decline of recent seabirds inferred from a composite 1000-year record of population dynamics. *Sci. Rep.* **6**, 35191; doi: 10.1038/srep35191 (2016).

## Supplementary Material

Supplementary Information

## Figures and Tables

**Table 1:** Results of radiocarbon analysis for bone samples from the three profiles.

Laboratory No	Sample No	Depth (cm)	Conventional Age BP	Cal BP (2σ)	Lab
293508	CH1-26	37	620 ± 30	226–414	Beta Analytic
13880	CH1-38	55	740 ± 20	318–473	U of Georgia
13884	CH1-50	72	1260 ± 30	740–911	U of Georgia
21474	ZS2-14	13.5	500 ± 20	56–256	U of Georgia
21475	ZS2-23	22.5	710 ± 20	300–449	U of Georgia
21476	ZS2-32	31.5	980 ± 20	530–641	U of Georgia
21477	ZS2-42	43	1030 ± 20	554–670	U of Georgia
21478	ZS2-51	61	1040 ± 20	558–677	U of Georgia
21479	ZS2-58	75	1150 ± 20	660–778	U of Georgia
21456	BD1-32	31.5	560 ± 20	134–286	U of Georgia
21457	BD1-43	45	730 ± 20	311–464	U of Georgia
21458	BD1-52	63	760 ± 20	352–488	U of Georgia
21459	BD1-62	83	930 ± 20	503–617	U of Georgia
21461	BD1-74	107	1320 ± 20	799–953	U of Georgia
21462	BD1-81	130	1330 ± 20	810–966	U of Georgia

**Table 2:** Basic characteristics of the three islands and sediment profiles.

Island	Core Location	Height above sea level (m)	Length × width (m × m)	Area (km^2^)	Profile code	Core length (cm)
Chenhang Island	16°27′10.1″N,111°42′34.0″E	5	315 × 1050	0.33	CH	72
Zhaoshu Island	16°58′39.9″N,112°16′15.5″E	4.4	700 × 400	0.20	ZS2	94
Beidao Island	16°57′45.0″N,112°18′38.5″E	8.2	1400 × 250	0.26	BD1	137

## References

[b1] Anderson W. B. & Polis G. A. Nutrient fluxes from water to land: seabirds affect plant nutrient status on Gulf of California islands. Oecologia 118, 324–332 (1999). 10.1007/s004420050733 28307276

[b2] Blais J. M. *et al.* Arctic seabirds transport marine-derived contaminants. Science 309, 445 (2005). 16020729 10.1126/science.1112658

[b3] Stempniewicz L., Błachowiak-Samołyk K. & Węsławski J. M. Impact of climate change on zooplankton communities, seabird populations and arctic terrestrial ecosystem-a scenario. *Deep-Sea Res*. Part II 54, 2934–2945 (2007).

[b4] Michelutti N. *et al.* Trophic position influences the efficacy of seabirds as metal biovectors. Proc. Natl. Acad. Sci. 107, 10543–10548 (2010). 20498048 10.1073/pnas.1001333107 PMC2890848

[b5] Ellis J. C. Marine birds on land: a review of plant biomass, species richness, and community composition in seabird colonies. Plant Ecol. 181, 227–241 (2005).

[b6] Devney C. A., Short M. & Congdon B. C. Sensitivity of tropical seabirds to El-Niño precursors. Ecology 90, 1175–1183 (2009). 19537539 10.1890/08-0634.1

[b7] Sun L. G. *et al.* Vertebrate records in polar sediments: biological responses to past climate change and human activities. Earth-Sci. Rev. 126, 147–155 (2013).

[b8] Nie Y., Sun L., Liu X. & Emslie S. D. From warm to cold: migration of Adélie penguins within Cape Bird, Ross Island. Sci. Rep. 5, 11530, doi: 10.1038/srep11530 (2015). 26113152 PMC4650636

[b9] Lu Y. C., Yang X. C. & Jia R. F., 1979. Quaternary biological sediments in the Xisha Archipelago, China and a discussion on the age of island-formation. Geochimica 2, 93–102 (in Chinese with English abstract) (2002).

[b10] Hainan Ocean Administration. The professional proceedings of the integrated investigation research on sea islands resource of Hainan province. (China Ocean Press, 1999) (in Chinese with English abstract).

[b11] Xu L. Q. *et al.* Geochemical evidence for the development of coral island ecosystem in the Xisha Archipelago of South China Sea from three ornithogenic sediment profiles. Chem. Geol. 286, 135–145 (2011).

[b12] Braje T. J. & Erlandson J. M. Human acceleration of animal and plant extinctions: A late Pleistocene, Holocene, and Anthropocene continuum. Anthropocene 4, 14–23 (2013).

[b13] Seddon A. W. *et al.* Looking forward through the past: identification of 50 priority research questions in palaeoecology. J. Ecol. 102, 256–267 (2014).

[b14] Exploration Group of Xisha Islands of Institute of Soil Science of Chinese Academy of Sciences (CAS). Soil and guano phosphorus mine in Xi-Sha. (Science Press, 1977). (in Chinese).

[b15] Cao L., Pan Y. L. & Liu N. F. Waterbirds of the Xisha Archipelago, South China Sea. Waterbirds 30, 296–300 (2007).

[b16] Cao L. Population ecology of the red-footed booby on the Xisha Archipelago. PhD dissertation. Lanzhou University (2005).

[b17] Tang S. The biodiversity research on a tropical coral archipelago in the South China Sea. Master dissertation. University of Science and Technology of China (2009).

[b18] Zhao H. T. *et al.* Nature and development of Yongxing island and Shi island of Xisha islands. Mar. Sci. Bull. 12, 44–56, (in Chinese with English abstract) (1994).

[b19] Blaauw M. & Christen J. A. Flexible paleoclimate age-depth models using an autoregressive gamma process. Bayesian Anal. 6, 457–474 (2011).

[b20] Appleby P. G. Three decades of dating recent sediments by fallout radionuclides: a review. Holocene 18, 83–93 (2008).

[b21] Reimer P. J. *et al.* IntCal13 and Marine13 radiocarbon age calibration curves 0-50,000 yr cal BP. Radiocarbon 55, 1869–1887 (2013).

[b22] Xu L. Q. *et al.* Distribution of radionuclides in the guano sediments of Xisha Islands, South China Sea and its implication. J. Environ. Radioact. 101, 362–368 (2010). 20346552 10.1016/j.jenvrad.2010.02.004

[b23] Liu X. D., Sun J., Sun L. G., Liu W. Q. & Wang Y. H. Reflectance spectroscopy: a new approach for reconstructing penguin population size from Antarctic ornithogenic sediments. J. Paleoliminol. 45, 213–222 (2011).

[b24] Xu L. Q., Liu X. D., Sun L. G. & Liu W. Q. Rapid identification of source material levels in coral sand ornithogenic sediments by reflectance spectroscopy. Ecol. Indicat. 23, 517–523 (2012).

[b25] Sun L. G., Xie Z. Q. & Zhao J. L. A 3,000-year record of penguin populations. Nature 407, 858 (2000). 11057656 10.1038/35038163

[b26] Wu L. B. *et al.* Dietary change in seabirds on Guangjin Island, South China Sea, over the past 1200 years inferred from stable isotope analysis. Holocene, doi: 10.1177/0959683616660163 (2016).

[b27] Cao L., Pang Y. L. & Liu N. F. Status of the red-footed booby on the Xisha Archipelago, South China Sea. Waterbirds 28, 411–419 (2005).

[b28] Moberg A., Sonechldn D. M., Holmgren K., Datsenko N. M. & Karlen W. Highly variable Northern Hemisphere temperatures reconstructed from low- and high-resolution proxy data. Nature 433, 613–617 (2005). 15703742 10.1038/nature03265

[b29] Yang B., Braeuning A. & Shi Y. A. General characteristics of temperature variation in China during the last two millennia. Geophys. Res. Lett. 29, 1324, doi: 10.1029/2001GL014485 (2002).

[b30] Huang T., Sun L. G., Wang Y. H., Liu X. D. & Zhu R. B. Penguin population dynamics for the past 8500 years at Gardener Island, Vestfold Hills. Antarct. Sci. 21, 571–578 (2009).

[b31] Emslie S. D., Coats L. & Licht K. A 45,000 yr record of Adélie penguins and climate change in the Ross Sea, Antarctica. Geology 35, 61–64 (2007).

[b32] Clucas G. V. *et al.* A reversal of fortunes: climate change ‘winners’ and ‘losers’ in Antarctic Peninsula penguins. Sci. Rep. 4, 5024, doi: 10.1038/srep05024 (2014). 24865774 PMC4034736

[b33] Younger J. L. *et al.* Too much of a good thing: Sea ice extent may have forced emperor penguins into refugia during the last glacial maximum. Glob. Change Boil. 21, 2215–2226 (2015). 10.1111/gcb.12882 25728986

[b34] Ropert-Coudert Y. *et al.* A complete breeding failure in an Adélie penguin colony correlates with unusual and extreme environmental events. Ecography 38, 111–113 (2015).

[b35] Liu K. K. *et al.* Monsoon-forced chlorophyll distribution and primary production in the South China Sea: observations and a numerical study. Dddp-Sea Res. Pt I 49, 1387–1412 (2002).

[b36] Shiozaki T. & Chen Y. L. Different mechanisms controlling interannual phytoplankton variation in the South China Sea and the western North Pacific subtropical gyre: A satellite study. Adv. Space Res. 52, 668–676 (2013).

[b37] Wei G. J. *et al.* Coralline Sr/Ca and Mg/Ca thermometer for the northern South China Sea: calibration and primary application on high resolution SST reconstructing. Quaternary Sci. 24, 325–331 (2004).

[b38] Song S. H. *et al.* Variation of the winter monsoon in South China Sea over the past 183 years: Evidence from oxygen isotopes in coral. Global Planet. Change 98, 131–138 (2012).

[b39] Zhao H. & Tang D. L. Effect of 1998 El Niño on the distribution of phytoplankton in the South China Sea. J. Geophy. Res. 112, doi: 10.1029/2006JC003536 (2007).

[b40] Meeker L. D. & Mayewski P. A. A 1400-year high-resolution record of atmospheric circulation over the North Atlantic and Asia. Holocene 12, 257–266 (2002).

[b41] Zhang P. Z. *et al.* A test of climate, sun and culture relationships from an 1810-year Chinese cave record. Science 322, 940–942 (2008). 18988851 10.1126/science.1163965

[b42] Yan H. *et al.* South China Sea hydrological changes and Pacific Walker Circulation variations over the last millennium. Nat. Commun. 2, 293, doi: 10.1038/ncomms1297 (2011). 21522137 PMC3104522

[b43] Yan H. *et al.* A record of the Southern Oscillation Index for the past 2,000 years from precipitation proxies. Nat. Geosci. 4, 611–614 (2011).

[b44] Mann M. E., Cane M. A., Zebiak S. E. & Clement A. Volcanic and solar forcing of the tropical pacific over the past 1000 years. J. Climate 18, 447–456 (2005).

[b45] Zhao H. T. History of expeditions to Xisha Islands. Geographical Research 15, 55–65 (in Chinese with English abstract) (1996).

[b46] Sun Y. *et al.* Strontium contents of a Porites coral from Xisha Island, South China Sea: A proxy for sea-surface temperature of the 20th century. Paleoceanography 19, PA2004, doi: 10.1029/2003PA000959 (2004).

[b47] Peng Z. C. *et al.* Coral δ^18^O records as an indicator of winter monsoon intensity in the South China Sea. Quaternary Res. 59, 285–292 (2003).

[b48] Liu Y. *et al.* The decline of winter monsoon velocity in the South China Sea through 20^th^ centrry: evidence from the Sr/Ca records in corals. Global Planet. Change 63, 79–85 (2008).

[b49] Chiu H. & Park C. H. Legal status of the Paracel and Spratly Islands. Ocean Dev. Int. Law 3, 1–28 (1975).

[b50] Wang Y., Ma Y. J., Yang Z. Z., Zheng J. Q. & Yu T. F. Identification of rodents and blood-sucking insects in Xisha Islands of China and the first report of Anopheles mosquitoes and midges. Acad. J. Second Mil. Med. Univ. 35, 581–585 (in Chinese with English abstract) (2014).

[b51] Yu K. F. Coral reefs in the South China Sea: Their response to and records on past environmental changes. Sci. China Ser. D-Earth Sci. 55, 1217–1229 (2012).

[b52] Wang D. R., Wu Z. J., Li Y. C., Chen J. R. & Chen M. Analysis on variation trend of coral reef in Xisha. Acta Ecol. Sin. 31, 254–258 (2011).

[b53] Myers R. A. & Worm B. Rapid worldwide depletion of predatory fish communities. Nature 423, 280–283 (2003). 12748640 10.1038/nature01610

[b54] Stobutzki I. C., Silvestre G. T. & Garces L. R. Key issues in coastal fisheries in South and Southeast Asia outcomes of a regional initiative. Fish. Res. 78, 109–118 (2006).

[b55] Christensen V., Garces L. R., Silvestre G. T. & Pauly D. Fisheries impact on the South China Sea Large Marine Ecosystem: a preliminary analysis using spatially explicit methodology. Assessment, Management and Future Directions for Coastal Fisheries in Asian Countries [Silvestre G. T. *et al.* (eds.)] 51–62 (World Fish Center Conference Proceedings 67, 2003).

[b56] Xie Z. Q. *et al.* Preliminary geochemical evidence of groundwater contamination in coral islands of Xi-Sha, South China Sea. Appl. Geochem. 20, 1848–1856 (2005).

[b57] Liu X. D. *et al.* Historical change of mercury pollution in remote Yongle archipelago, South China Sea. Chemosphere 87, 549–556 (2012). 22284978 10.1016/j.chemosphere.2011.12.065

[b58] Xu L. Q. *et al.* A 700-year record of mercury in avian eggshells of Guangjin Island, South China Sea. Environ. Pollut. 159, 889–896 (2011). 21262554 10.1016/j.envpol.2010.12.021

[b59] Rawlence N. J. *et al.* Geographically contrasting biodiversity reductions in a widespread New Zealand seabird. Mol. Ecol. 24, 4605–4616 (2015). 26227633 10.1111/mec.13338

