# Supplementary material for: Decline of recent seabirds inferred from a composite 1000-year record of population dynamics
Source: Sci Rep. 2016 Oct 17;6:35191. doi: 10.1038/srep35191 (PMC5066250; doi:10.1038/srep35191)
Supplement: Supplementary Information [file srep35191-s1.pdf]

Supplementary materials

Decline of recent seabirds inferred from a composite 1000-year record of population dynamics

Liqiang Xu[1,2 *] Xiaodong Liu[2 *], Libin Wu[2], Liguang Sun[2], Jinjun Zhao[1], Lin Chen[3]

1 School of Resources and Environmental Engineering, Hefei University of Technology, Hefei, Anhui 230009, China

2 Institute of Polar Environment, School of Earth and Space Sciences, University of Science and Technology of China, Hefei, Anhui 230026, China

3 Xisha Deep Sea Marine Environment Observation and Research Station, South China Sea Institute of Oceanology, Chinese Academy of Sciences, Sansha, Hainan 573199, China

* Correspondence and requests for materials should be addressed to: L.X (xlq@hfut.edu.cn) or X.L (ycx@ustc.edu.cn)

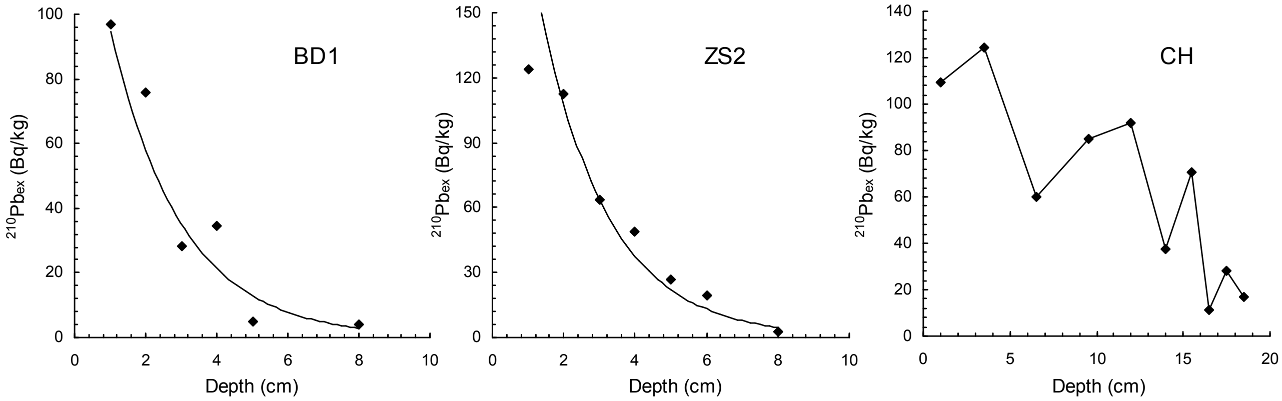

Supplementary Fig. S1 **Concentration-versus-depth profiles of excess ^{210}Pb for the surface sediments of profiles BD1, ZS2 and CH**

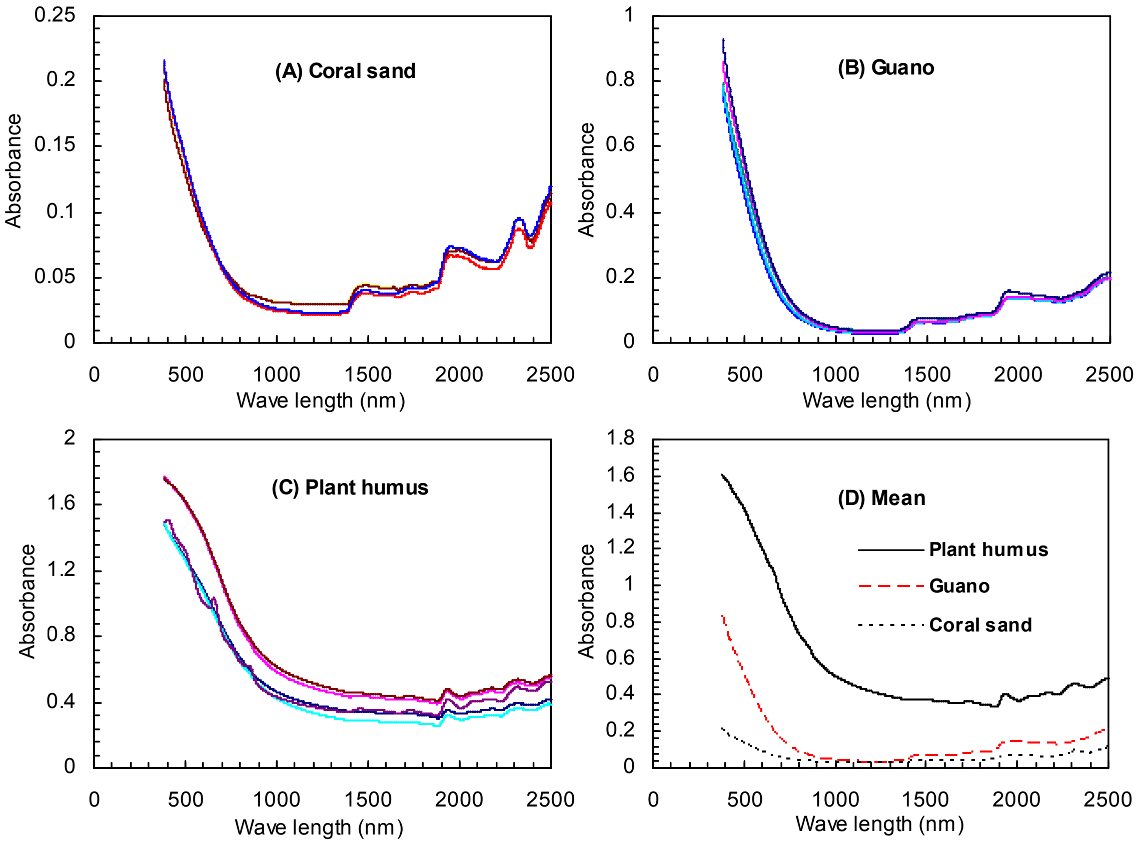

Supplementary Fig. S2 Characteristic spectra of three end members. A, B and C are spectra of coral, guano and plant humus, respectively, and D is the mean of each. [Reprinted from Ecological Indicators, 23, Xu, L.Q., Liu, X.D., Sun, L.G., & Liu, W.Q. Rapid identification of source material levels in coral sand ornithogenic sediments by reflectance spectroscopy, 517-523, Copyright (2012), with permission from Elsevier.]

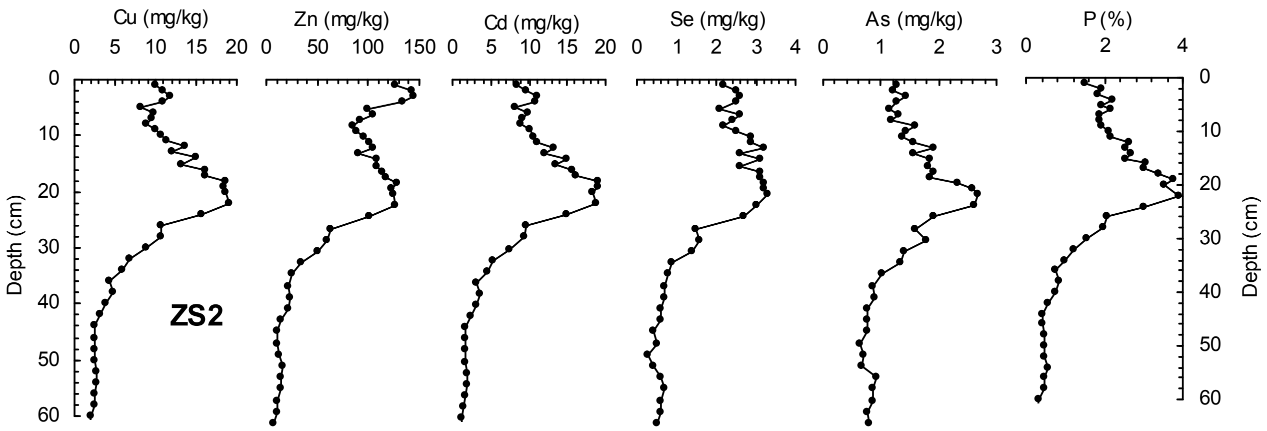

Supplementary Fig. S3 Concentration-versus-depth profiles of avian bio-elements for the profile ZS2

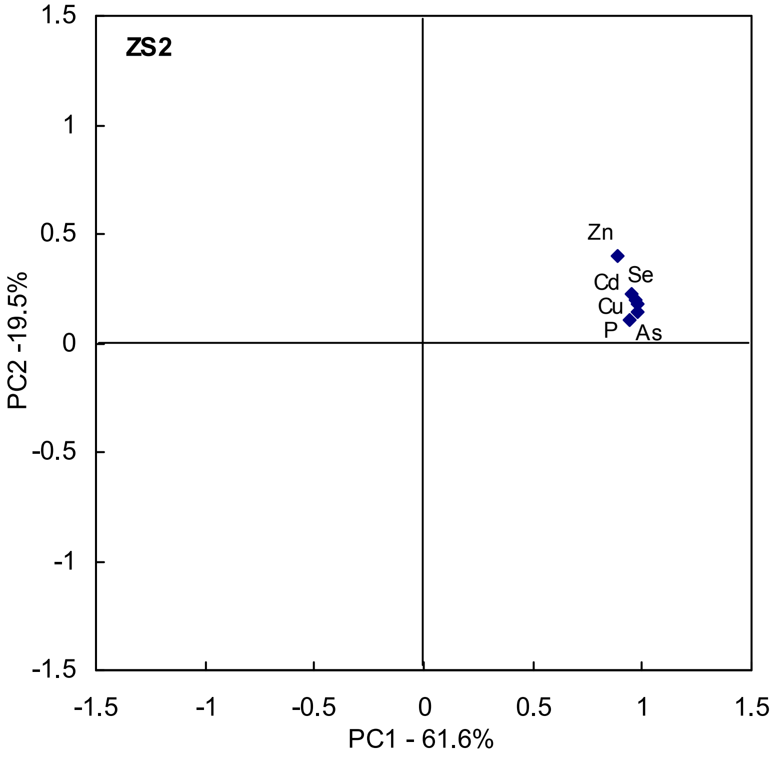

Supplementary Fig. S4 PCA variables for bio-elements in the sediment core ZS2
